# The Role of Spectacle Lenses in the Control and Management of Myopia Progression: A Narrative Review

**DOI:** 10.3390/life15091415

**Published:** 2025-09-08

**Authors:** Livio Vitiello, Filippo Lixi, Valerio Calabresi, Mario Troisi, Ilaria De Pascale, Alfonso Pellegrino, Giulia Coco, Aldo Vagge, Giuseppe Giannaccare

**Affiliations:** 1 Eye Unit, “Luigi Curto” Hospital, Azienda Sanitaria Locale Salerno, 84035 Polla, Italy; livio.vitiello@gmail.com (L.V.); idepascale@outlook.it (I.D.P.); al.pellegrino@aslsalerno.it (A.P.); 2 Eye Clinic, Department of Surgical Sciences, University of Cagliari, 09124 Cagliari, Italy; valerio.calabresi1@gmail.com (V.C.); giuseppe.giannaccare@gmail.com (G.G.); 3 Eye Clinic, Department of Neurosciences, Reproductive and Odontostomatological Sciences, University of Naples Federico II, 80131 Naples, Italy; troisi165@gmail.com; 4 Ophthalmologic Unit, University Hospital of Salerno, 84100 Salerno, Italy; 5 Ophthalmology Unit, Department of Clinical Sciences and Translational Medicine, University of Rome Tor Vergata, 00133 Rome, Italy; giulia.coco@uniroma2.it; 6 Eye Unit, IRCCS Ospedale Policlinico San Martino, 16132 Genoa, Italy; aldo.vagge@unige.it; 7 DINOGMI, University of Genoa, 16132 Genoa, Italy

**Keywords:** CARE lenses, DIMS lenses, HALT lenses, myopia, myopia control, myopia progression, spectacle lenses

## Abstract

The number of people affected by myopia worldwide is estimated to reach two billion and to further grow. Therefore, numerous treatment approaches (pharmacological, behavioral, environmental, and optical) have been put forth to slow the progression of myopia, especially in children. Among these, spectacle lenses represent a straightforward and less intrusive therapeutic approach for children and their parents. For this reason, in recent years, several spectacle lenses with different technologies have been developed to slow myopia progression and enhance the quality of life for myopic children, thus trying to reduce the related health care burden. According to the published scientific literature, three different types of spectacle lenses are currently the most validated optical options for myopia management: (i) Defocus Incorporated Multiple Segments lenses (DIMS), (ii) Highly Aspherical Lenslet Target lenses (HALT) and (iii) Cylindrical Annular Refractive Elements (CARE) lenses. The aim of this narrative review is to exclusively discuss the scientific evidence of these three different spectacles lenses, and to point out the potential benefits and drawbacks in their use for myopia control and management.

## 1. Introduction

Globally, myopia has become a serious issue that cannot be disregarded. The number of people with myopia is predicted to increase from 2 billion to 5 billion by 2050 [1]. For this reason, myopia is currently seen as a significant health care burden. Specifically, the annual prevalence of myopia that begins between the ages of 7 and 15 is continuously rising, underscoring the necessity for standardized methods to manage its progression and prevent potentially permanent consequences like myopic maculopathy, glaucoma, retinal detachment, and cataract formation [2]. Therefore, to preserve eye health and enhance quality of life, an effective strategy to slow the progression of myopia is essential.

After a child has been diagnosed with myopia, a thorough treatment plan should be established. Age of onset, baseline refractive status, visual surroundings, familiar compliance and history, risks and benefits of the therapy, and annual cost are all aspects that should be considered [3,4]. Reducing the progression of myopia once it has started is the primary therapeutic goal, irrespective of the therapeutic method [1,5]. Numerous strategies have been investigated, such as contrast-attenuation filters [6], environmental control [7], pharmaceutical drops [8], and other types of visual therapeutic options [9,10]. In addition, combination therapies, including the concomitant use of spectacle lenses and low-dose atropine, have shown enhanced efficacy in recent studies [11], while developing individualized treatment algorithms based on patient-specific response patterns represents a promising direction for future research.

For children under the age of 8, the use of spectacle lenses is a simple and less invasive method [4]. However, they are also the most fundamental approach in providing clear vision and there is evidence suggesting that myopia can worsen more quickly if spectacles are not worn regularly. For example, a public health policy paper by Yap and Mishu [12] highlighted that under- or uncorrected myopia can lead to a vicious circle of myopia progression and this can even disproportionately affect low-income families and widen health inequality. For this reason, in recent years, several spectacle lenses have been developed with specific technologies that have been shown to be able to reduce the progression of myopia, thus improving the quality of life of myopic children [13].

From an optical perspective, uncorrected myopia results in central retinal image formation anterior to the retina, while the peripheral wavefront may partially focus posterior to the retina, creating a hyperopic defocus. Conventional single vision (SV) spectacle lenses correct refractive errors uniformly across the central and peripheral retina. While this correction shifts the central focal plane onto the retina, the peripheral focal plane remains posterior to the retina, thereby inducing peripheral hyperopic defocus, an optical condition that may stimulate axial elongation and contribute to myopia progression [14]. In contrast, the introduction of myopic defocus (MD), achieved through the addition of relative positive power in the peripheral visual field, has been shown in various animal models to suppress axial eye growth. Across different species and experimental designs, a consistent finding emerged: the application of MD, whether added to a hyperopic or plano correction, significantly reduced ocular elongation compared to control animals or fellow untreated eyes [15,16,17].

The purpose of this narrative review is to provide an updated analysis of the published literature focused solely on the benefits and the results of the spectacle lenses currently available for myopia management, namely Defocus Incorporated Multiple Segments (DIMS) lenses, Highly Aspherical Lenslet Target (HALT) lenses and Cylindrical Annular Refractive Elements (CARE) lenses.

In addition, this narrative review will provide an up-to-date comparison of the discussed spectacle lenses, to ensure a greater awareness among clinicians of these therapeutic aids currently available for managing myopia in children, thus trying to halt its progression.

## 2. Materials and Methods

A wide-ranging literature search was performed on PubMed, Google Scholar, and Scopus databases using the following search terms: “myopia” OR “myopia management” OR “myopia control” OR “myopia progression” AND words related to the three different main validated types of spectacle lenses utilized for the control of myopia in children, such as “Defocus Incorporated Multiple Segments lenses” OR “DIMS lenses”, “Highly Aspherical Lenslet Target lenses” OR “HALT lenses” and “Cylindrical Annular Refractive Elements lenses “ OR “CARE lenses”.

The search was performed in August 2025, and only the English research articles exclusively regarding the use of spectacle lenses for myopia control in children were included in this narrative review. On the other hand, full research articles considering other therapeutic approaches (such as low-dose atropine, contact lenses and orthokeratology), the combination of these therapeutic strategies with the spectacle lenses, and duplicate papers were excluded. The reference lists of the included studies were also manually reviewed to find any new publications that could be pertinent to the discussed topic.

The initial bibliographic search yielded 151 results for DIMS lenses, 36 results for HALT lenses, and 6 results for CARE lenses. At last, 18 papers on DIMS lenses, 10 papers on HALT lenses, and 4 papers on CARE lenses were included in this review.

In addition, four additional papers comparing these types of spectacle lenses were also added to this review.

## 3. Defocus Incorporated Multiple Segments Lenses

In 2018, a new type of spectacle lens utilizing peripheral defocus technology, called DIMS, has been released in the market under the brand name MiyoSmart^®^ (Hoya Lens, Tokyo, Japan) [18]. The DIMS lens design is based on MD in the peripheral retina, thus mitigating axial eye growth [14]. The lens comprises two distinct optical zones: a central optical zone with a 9 mm diameter for correcting distance refractive errors, and a mid-peripheral “treatment zone” (approximately 33 mm in diameter) composed of a honeycomb structure with 396 microlens segments (each 1.03 mm in diameter), providing a relative positive power of +3.50 diopters (D). This configuration simultaneously delivers MD to the peripheral retina, while maintaining undistorted central visual acuity across all distances. The peripheral MD creates multiple focal points anterior to the retinal plane, which are perceived as blurred images by the retina, thereby inhibiting axial eye growth [18]. The lens is manufactured from polycarbonate material with a refractive index of 1.590 and features a multi-coating that minimizes surface reflections. It is also water-repellent, preventing liquid accumulation on the lens surface and enhancing visual comfort and durability [18]. DIMS lenses can correct myopia up to −6.50 D and myopic astigmatism up to −4.00 D. Additionally, the lenses allow a prismatic correction up to 3.00 D per lens [18,19].

Several recent studies have evaluated the use and visual impact of DIMS lenses. Lu and colleagues firstly assessed the acceptability and adaptability of this lens type in a prospective cross-over study involving 20 Chinese children, who were randomly assigned to wear both DIMS and SV lenses [20]. Distance visual acuity (VA) in the primary gaze was assessed under both standard and dim lighting conditions. Measurements were taken before and after 30 min of lens wearing for both DIMS and SV lenses. Additionally, VA at approximately 40 cm within the mid-peripheral visual field was evaluated under both illumination levels. Central VA remained unaffected by the DIMS lenses compared to SV lenses under all testing conditions. Differently, near mid-peripheral VA was reduced by approximately 0.06 logarithm of the minimum angle of resolution in two out of four quadrants under standard illumination, and in three quadrants under dim illumination. Nonetheless, being aware of the average anti-myopic efficacy, 90% of children preferred DIMS lenses [20].

Lam et al. released the first results on the anti-myopic effect of DIMS lenses in a 2-year, double-masked randomized controlled trial on 183 Chinese children aged 8 to 13 years. Participants were randomly assigned to wear either DIMS lenses or SV spectacle lenses. The results demonstrated that children in the DIMS group exhibited a 52% slower rate of myopic progression compared to those in the SV group. In addition, axial length (AL) elongation was 62% lower in the DIMS group, with a mean intergroup difference of 0.34 mm. Notably, over the two-year study follow-up, 21.5% of children in the DIMS group experienced no progression of myopia, in contrast to only 7.4% in the SV group [19].

In a subsequent analysis of the same cohort, Zhang et al. evaluated relative peripheral refraction (RPR) across horizontal retinal eccentricities. After two years, children in the DIMS group exhibited a symmetrical peripheral myopic shift across the horizontal retina, which correlated with significantly reduced axial elongation and slower myopia progression. In contrast, the SV group showed asymmetric RPR changes, with a significant hyperopic shift observed in the nasal retina [21].

In the third year of the study, children who had initially worn SV lenses were switched to DIMS lenses and compared with those who had worn DIMS lenses continuously for three years. The latter group maintained effective myopia control and a stable, symmetrical RPR profile without significant changes. Similarly, the Control-to-DIMS group demonstrated significant lower myopia progression following the introduction of DIMS lenses in the third year [22].

Moreover, the relationship between baseline RPR and subsequent changes in myopia and AL was also investigated [23]. Within the DIMS group, children presenting with baseline myopic RPR experienced significantly greater myopia progression and axial elongation compared to those with baseline hyperopic RPR. This association was not observed in the SV group, where baseline RPR had no predictive value for future myopia progression or AL changes [23].

Based on the cohort of a previous randomized controlled trial [19], a further analysis was carried out to investigate the effect of DIMS lenses on subfoveal choroidal thickness over a period of two years. A significant increase in this parameter was observed as early as one week following DIMS lens wearing, with this thickening maintained throughout the study duration [24]. Furthermore, choroidal changes at three months demonstrated to help the prediction of AL changes after one year [25]. The potential role of the choriocapillaris in predicting myopia progression was further investigated, showing that reduced choriocapillaris flow may be associated with more rapid myopia progression [25].

The research group led by Lam carried out additional studies to evaluate visual function in children wearing DIMS lenses and to extend the follow-up of their initial findings to 3 and 6 years [26,27,28]. No significant differences in visual function were observed between the DIMS and SV groups after two years of lens wearing. Specifically, both groups exhibited statistically significant reductions in accommodative lag and both monocular and binocular amplitudes of accommodation. However, no significant changes were noted in distance low-contrast VA, near high-contrast VA, near low-contrast VA, or phoria [26].

In the three-year follow-up study, three groups were assessed: (i) children who had continuously worn DIMS lenses, (ii) children who switched from SV to DIMS lenses after two years, and (iii) a historical control group of age-matched children who did not use DIMS lenses [27]. Over the third year, changes in spherical equivalent refraction (SER) and AL in the DIMS group were not statistically significant, indicating myopia stabilization. The Control-to-DIMS group exhibited reduced myopia progression and axial elongation compared to their first and second years. In addition, both the DIMS and Control-to-DIMS groups showed significantly less progression in SER and AL than the historical control group [27].

At the 6-year follow-up, DIMS lenses continued to demonstrate long-term efficacy in myopia control without associated adverse effects [28]. Children who had worn DIMS lenses throughout the study period showed significantly lower myopia progression and axial elongation than those in the SV group, with no evidence of a rebound effect following treatment discontinuation [28]. It is also worth noting that, beyond the proven efficacy in controlling myopia, DIMS lenses showed a clinically significant axial shortening after more than 2 years of lens wearing in a small proportion of patients (2.7%) [29].

Recently, the first results on the efficacy of DIMS lenses in European clinical settings have been published. Data from a retrospective analysis performed in a real-life clinical setting in Germany showed that, after 12 months of treatment, more than 64% of participants experienced no or only minimal myopia progression, and over 45% of eyes exhibited AL growth within the physiological range. Moreover, children older than 10 years with an AL below the 98th percentile at baseline were more likely to respond successfully to treatment compared to younger children with higher baseline AL values [30]. Similar findings were also reported in a 3-year retrospective study carried out in Italy, where DIMS lenses effectively slowed myopia progression in pediatric patients, with more favorable outcomes observed in children older than 10 years [31]. Additionally, Domsa and colleagues identified several risk factors associated with suboptimal treatment response, including younger age, astigmatism, and the presence of high myopia in the mother [32].

Despite their overall safety profile, some concerns remain regarding the potential impact of DIMS lenses on visual function and comfort. While different studies have reported that DIMS lenses are generally safe from a visual standpoint, showing no significant alterations in standard visual parameters [26] or visual cortex responses compared to SV lenses [33], other investigations have identified changes in binocular vision and accommodative function following 24 months of DIMS lens wearing [34], consistent with earlier findings by Lam and colleagues [26]. Additionally, eye strain, peripheral blur, headaches, and halos have been reported, particularly during the initial adaptation period [32,35]. Nevertheless, participants reported high levels of satisfaction in quality of life, including social relationships, physical well-being, and psychological health [35].

Finally, a recent study found no significant differences in the Quality of Life Impact of Refractive Correction questionnaire scores between DIMS and SV lens wearers, suggesting that DIMS lenses can provide a vision-related quality of life comparable to that one of conventional SV lenses [36].

Table 1 summarizes the main clinical research studies concerning the use of DIMS lenses in children.

## 4. Highly Aspherical Lenslet Target Lenses

Since 2020, spectacle lens that utilizes HALT technology was first launched in Canada by Essilor under the name of Stellest^®^ (Essilor, Charenton-le-Pont, France). HALT lenses feature a clear central optical zone for accurate distance vision, encircled by a treatment zone of hundreds of high plus aspherical lenslets. These small lenslets are meticulously arranged in concentric rings and engineered to generate a three-dimensional “volume of myopic defocus” over the mid-to-peripheral retina. This sustained myopic defocus aims to counteract axial elongation while preserving central visual acuity [37].

The efficacy of HALT lenses in reducing SER progression and AL was first demonstrated in a two-year randomized controlled trial by Bao et al. in Chinese children aged 8–13 years [37]. HALT lenses reduced SER progression by 0.80 ± 0.11 D (67% reduction) and AL elongation by 0.35 mm (64%) compared to SV lenses. Notably, in children with at least 12 h of lenses use, HALT achieved an even greater effect, with a SER reduction of 0.99 D (67%) and an AL reduction of 0.41 mm (60%) [37].

These findings were corroborated in another randomized controlled trial by the same group comparing HALT, slightly aspherical (SA) lenses and SV lenses in 170 children. The SV lenses group showed SER progression of –0.81 ± 0.06 D and AL elongation of 0.36 ± 0.02 mm. HALT lenses reduced SER progression by 0.53 D (67%) and AL elongation by 0.23 mm (64%), while SA lenses achieved 0.33 D (41%) and 0.11 mm (31%) reductions, respectively. HALT outperformed SA lenses significantly in both SER and AL, despite equivalent best-corrected VA and wearing time across groups, and no adaptation issues or adverse events reported [38].

Further evidence came from a 12-month, double-blind, crossover randomized controlled trial involving 119 Vietnamese children, performed by Sankaridurg and co-authors. In the first 6 months, HALT slowed SER progression and significantly reduced AL elongation compared to SV lenses. In the second phase, these differences became more pronounced, with no rebound effect reported when switching from HALT to SV lenses [39].

In addition, the long-term efficacy of HALT has been well documented. In a 4-year clinical trial HALT reduced SER progression by 1.34 D (54%) and AL elongation by 0.62 mm (52%) compared to the SV lenses control group [40]. These findings are consistent with the 5-year prospective study by Li et al., in which HALT slowed myopia progression by 1.27 ± 0.14 D versus 3.03 ± 0.18 D in the SV lenses group, and limited AL elongation to 0.72 ± 0.10 mm over five years, effectively preventing three years of progression [41].

Probably, HALT’s control over ocular growth appears linked to its effect on retinal shape and peripheral defocus. In a two-year trial, Huang et al. found that HALT lenses limited nasal peripheral eye elongation (especially at 30°) and induced the least negative shift in peripheral refraction among groups. While SA and SV lenses wearers exhibited a hyperopic shift in RPR, HALT wearers showed a less hyperopic profile, suggesting that HALT may contribute to a flatter retinal shape and mitigate peripheral hyperopic defocus [42].

Visual safety and performance have also been addressed. Gao et al. assessed visual field sensitivity in 21 adults using automated static perimetry. HALT lenses produced only minor differences compared to SV lenses, with a single significant increase (1.1 dB at 30° temporal, *p* < 0.00065), which was clinically irrelevant. No correlation with age or SER was found, suggesting HALT lenses preserve peripheral visual function [43].

Beyond myopia, Zhang evaluated HALT’s efficacy in low hyperopic children (6.0–9.9 years, SER 0.00 to +2.00 D). Although 1-year SER changes were like SV lenses, HALT lenses significantly reduced AL elongation, particularly in children wearing the lenses >30 h/week, reinforcing the role of compliance and extending HALT’s relevance to early use in non-myopic children [44]. Similar results were also obtained on non-myopic children by Wang and coauthors. In detail, HALT lenses were effective in slowing axial elongation and SER progression among non-myopic children aged 4–9 year [45]. Moreover, the duration of lens wearing was positively correlated with the reduction in axial elongation, suggesting a clear dose–response effect [45].

Finally, Wong et al. found that full-time HALT wearers (≥12 h/day, *n* = 96) had a mean AL increase of just 0.34 mm over two years in 157 Chinese children. Remarkably, ~90% of HALT users achieved axial growth rates similar to or slower than those expected for emmetropic children, suggesting that HALT may help normalize eye growth and not just reduce progression [46].

Table 2 shows the main clinical investigations performed on HALT lenses.

## 5. Cylindrical Annular Refractive Elements Lenses

In addition to DIMS and HALT lenses, cylindrical annular refractive elements have been included into a more recent design of spectacle lenses (MyoCare^®^, Zeiss Vision Care, Aalen, Germany). The lens has a myopia-correcting center optical zone that is surrounded by a treatment zone with many micro-cylinders grouped in concentric rings. These 0.5 mm wide circular cylindrical refractive elements alternate with equally wide annular zones that share the distance-correcting optic’s refractive characteristics. The radial and circumferential powers of the cylindrical annuli are +9.2 D and 0 D, respectively, resulting in an average cylindrical power of +4.6 D. The alternating cylindrical elements in conjunction with the clear zones are considered to induce simultaneous defocus at the retina [47].

Liu and colleagues evaluated 96 Chinese children aged 8–12 years with −1.00 D to −4.00 D of spherical component myopia and <1.50 D astigmatism, which were randomly assigned to wear CARE or SV spectacle lenses [48]. The authors found that CARE lenses significantly reduced the rate of axial elongation over 1 year compared with SV lenses, also reducing the myopia progression.

Similarly, Chen and colleagues demonstrated the efficacy of CARE lenses in reducing myopia progression and axial elongation in the same cohort of 6–13 year-Chinese children in a period of one year [49] and two years [50] compared to SV lenses.

The only clinical study on CARE lenses performed on European children was carried out by Alvarez-Peregrina et al. over a period of one year [51]. This study confirmed that children wearing CARE lenses showed less myopia progression compared to SV lenses [51].

Finally, in all the discussed studies, children adapted to their lenses with no reported adverse events, complaints, or discomfort.

Table 3 summarizes the main findings of the clinical studies performed on CARE lenses.

## 6. DIMS vs. HALT vs. CARE Lenses

A direct comparison between HALT and DIMS lenses was first made by Guo and colleagues [52]. In a retrospective cohort study involving 257 Chinese children, they found that HALT led to significantly less SER progression and AL elongation than DIMS over one year, even after adjusting for baseline parameters [52].

In a real-world study of the French Myopia Cohort, Najji and colleagues compared children using SV lenses with participants using either DIMS or HALT lenses [53]. On a total of 7626 children, both DIMS and HALT lenses demonstrated efficacy in reducing myopia progression compared with SV lenses. While a statistically significant lower myopia progression rate was observed in the HALT group, this difference was not clinically significant [53].

Lembo et al. performed a two-year retrospective cohort study comparing HALT and DIMS lenses. SER progression and axial elongation were similar between the two lenses, with a slightly higher but not statistically significant AL increase seen in DIMS lenses at 1 year. Interestingly, a higher proportion of DIMS wearers (38.4%) showed no SER progression at 2 years, compared to 21.9% in the HALT group [54].

So far, no prospective comparative studies have compared efficacy of all the three designs of lenses for preventing myopia progression. To address this gap, Gupta et al. have randomized 120 children to wear either DIMS, HALT or CARE spectacles full-time. Spectacle lenses incorporating peripheral defocus were all effective in reducing the rate of myopia progression significantly, with no adverse effects being observed. At the 1-year follow-up, the rate of myopia progression reduced by 0.38 ± 0.13 D/year (56.7%), 0.36 ± 0.12 D/year (58.1%) and 0.31 ± 0.15 D/year (47%) for the DIMS, HALT and CARE groups, respectively. The AL change was 0.2 ± 0.11 mm, 0.19 ± 0.12 mm and 0.23 ± 0.14 mm, respectively. Among the three designs, DIMS and HALT exhibited comparable and significantly better efficacy than CARE spectacles at 1-year follow-up [55].

Table 4 summarizes the main findings of the clinical studies comparing the three different spectacle lenses utilized for myopia control in children.

## 7. Conclusions

As the strategy of controlling myopia has gained more attention in recent years, spectacle lenses that permit a slowing in myopia progression with a good visual function have been developed. Daily use of these lenses has been demonstrated to successfully postpone the evolution of myopia and axial elongation in myopic children when compared to SV lenses. In particular, DIMS, HALT, and CARE lenses were all demonstrated to be effective in significantly reducing the rate of myopia progression, with no adverse effects reported. Among the three designs, DIMS and HALT demonstrated comparable efficacy, both outperforming CARE lenses at the 1-year follow-up.

Although spectacle lenses demonstrate sustained beneficial effects over extended follow-up periods (6–8 years), concerns regarding optimal treatment duration and long-term outcomes into adulthood remain unclear.

In fact, a critical analysis of the current literature reveals several limitations, considering that most studies have been conducted in East Asian populations, limiting the generalizability of results to other ethnic and environmental contexts.

Moreover, further concerns include methodological heterogeneity across studies in terms of inclusion criteria, definitions of progression, environmental controls, sample size and patient compliance, which creates a significant bias, making analytical synthesis challenging and limiting the robustness of indirect comparisons.

In conclusion, the discussed spectacle lenses have demonstrated significant efficacy as a therapeutic strategy for myopia control. However, further studies exploring their potential in pre-myopic children and longer follow-up would help to better define their full therapeutic promise.

## Figures and Tables

**Table 1:** Clinical investigations on myopia progression management with Defocus Incorporated Multiple Segments lenses.

Author (Year)	Duration	Type of Study	Population & Type of Lens	Assessment	Race	Age	Inclusion Criteria	Main Outcomes
***Lu et al. (2020)*** [20]	2 weeks	Prospective, cross-over study	20 children were recruited to wear both DIMS and SVL, with a random assignment.	High and low contrast central distant VA and high contrast mid-peripheral near VA were measured at both 500 lux and 50 lux ambient illuminance after 30 min’s and after a week’s wearing of the lens.	Chinese (Asian)	7–15 years	SER: −0.50 to −6.00 D; astigmatism of ≤1.50 D; interocular anisometropia of ≤1.25 D.	Central VA was not affected by DIMS lens compared with SVL. Near mid-peripheral VA was reduced in two out of four quadrants under standard illumination, and in three quadrants under dim illumination when wearing DIMS lenses. Mid-peripheral blurred vision was the main visual complaint, but 90% of children subjects preferred DIMS lenses.
***Lam et al. (2020)*** [19]	24 months	Double-masked randomized controlled trial	183 children were randomly assigned to wear DIMS (*n* = 93) or SVL (*n* = 90).	SER and AL were measured at 6-month intervals over 2 years.	Chinese (Asian)	8–13 years	Myopia between −1.00 and −5.00 D; astigmatism and anisometropia ≤ 1.50 D	Average myopic progressions over 2 years were −0.41 ± 0.06 D in the DIMS group and −0.85 ± 0.08 D in the SVL group. Mean axial elongation was 0.21 ± 0.02 mm and 0.55 ± 0.02 mm in the DIMS and SVL groups, respectively. Myopia progressed 52% more slowly for children in the DIMS group, while axial elongation by 62%. No myopia progression for 21.5% of children wearing DIMS lenses over 2 years, while only 7.4% for those ones wearing SVL.
***Zhang et al. (2020)*** [21]	24 months	Double-blind randomized controlled trial	183 children were allocated to either wearing DIMS (*n* = 93) or SVL (*n* = 90).	Peripheral refraction at 10°, 20°, and 30° of the nasal (10 N, 20 N, 30 N) and temporal (10 T, 20 T, 30 T) retinal eccentricities, central refraction, and axial length after cycloplegia were monitored every 6 months.	Chinese (Asian)	8–13 years	Myopia between −1.00 and −5.00 D; astigmatism and anisometropia ≤ 1.50 D	DIMS group showed more symmetrical peripheral myopic shifts and stable retinal peripheral refraction than SVL group, with also a slower axial elongation and a flatter retinal profile.
***Lam et al. (2020)*** [26]	24 months	Double-blind randomized controlled trial	160 myopic children were randomly assigned to wear DIMS (*n* = 79) or regular SVL (*n* = 81) full time for 2 years.	Visual function, including high-contrast VA and low-contrast VA at distance and near, binocular functions, and accommodation, before, during, and after 2 years of spectacle wear were assessed when both groups wore SVL corrections, also comparing changes of visual function between and within the two groups.	Chinese (Asian)	8–13 years	Myopia between −1.00 and −5.00 D; astigmatism and anisometropia ≤ 1.50 D	No statistically significant differences in the 2-year visual function changes between DIMS and SVL groups. Statistically significant improvement in the best-corrected distance high-contrast VA and stereoacuity score were found after DIMS lens wearing over 2 years. Similar findings were observed after SVL wear. For both the DIMS and SVL groups, there were statistically significant decreases in accommodative lag, monocular and binocular amplitude of accommodation after two years but not in the changes in distance low-contrast VA, near high-contrast VA, near low-contrast VA, or phoria.
***Lam et al. (2020)*** [27]	36 months	Prospective controlled trial (double-blind randomized in the first 2 years)	128 children who completed the 2-year randomized controlled trial were included The children who had worn DIMS lenses continued to wear DIMS lenses, while children who had worn SVL switched to wear DIMS lenses. Historical controls were used for comparing the third-year changes.	Cycloplegic SER and AL were measured at 6-month interval.	Chinese (Asian)	8–13 years	Myopia between −1.00 and −5.00 D; astigmatism and anisometropia ≤ 1.50	Over 3 years, DIMS group exhibited non-significant changes in SER and AL. In the Control-to-DIMS group, third-year changes in SER and AL were significantly smaller compared to both the first and second years. Changes in SER and AL in both groups over that period were significantly less than in the historical control group.
***Zhang et al. (2023)*** [22]	36 months	Prospective controlled trial (double-blind randomized in the first 2 years)	Children were randomly assigned to wear either the DIMS lens or SVL. After the 2-year randomized controlled trial, both groups were asked to continue for a further year, with the SVL group switched to DIMS lenses.	Central and peripheral refraction and AL were monitored every 6 months.	Chinese (Asian)	8–13 years	Myopia between −1.00 and −5.00 D; astigmatism and anisometropia ≤ 1.50	Over 3 years, the DIMS group (*n* = 65) showed good myopia control and maintained a relatively constant and symmetrical retinal peripheral refraction profile without significant changes. In the first 2 years, the SVL group (*n* = 55) showed asymmetrical retinal peripheral refraction changes, with significant increases in hyperopic retinal peripheral refraction. The Control-to-DIMS group showed significant myopia retardation after wearing DIMS lenses in the third year.
***Zhang et al. (2022)*** [23]	24 months	Double-masked randomized controlled trial	Children in the current study were participants in a 2-year randomized controlled trial. Data from 79 children and 81 children in the DIMS and SVL group were analyzed.	Peripheral refraction at 10°, 20°, and 30° nasal (10 N, 20 N, 30 N) and temporal (10 T, 20 T, 30 T) retina were measured at six-month intervals	Chinese (Asian)	8–13 years	Myopia between −1.00 and −5.00 D; astigmatism and anisometropia ≤ 1.50 D	In the DIMS group, greater baseline myopic retinal peripheral refraction spherical equivalent was associated with more myopic progression and greater axial elongation. In the SVL group, baseline retinal peripheral refraction had association only with myopia progression.
***Chun et al. (2023)*** [24]	24 months	Double-masked randomized controlled trial	158 Children in both DIMS and SVL groups were required to wear the assigned spectacle lenses in full-time mode.	Macular optical coherence tomography images from both eyes were collected at a similar time at baseline and different follow-up visits.	Chinese (Asian)	8–13 years	Myopia between −1.00 and −5.00 D; astigmatism and anisometropia ≤ 1.50	Subfoveal choroidal thickness increased significantly after one week of DIMS lens wear compared to those wearing SVL. The thickness of choroid increased to 13.64 ± 2.62 µm after 12 months of DIMS lens wear while the choroid thinned in SVL group (− 9.46 ± 2.55 µm). Choroidal thickening showed a significant negative association with axial elongation over two years in both the DIMS and SVL groups.
***Lam et al. (2023)*** [28]	72 months	Prospective controlled trial (double-blind randomized in the first 2 years)	Children who completed both the 2-year randomized controlled trial and the 3rd year study of DIMS spectacle lenses were invited to participate in this follow-up study of 6 years and divided into 4 groups. Group 1 wore DIMS spectacles from 0 to 6 years; Group 2 wore DIMS spectacles from 0 to 3.5 years and changed to wearing SVL afterwards; Group 3 wore SVL in the first 2 years and switched to DIMS spectacles afterwards; Group 4 wore SVL in the first 2 years, switched to wear DIMS spectacles for 1.5 years and then switched to SVL again.	Cycloplegic refractions and AL were measured.	Chinese (Asian)	8–13 years	Myopia between −1.00 and −5.00 D; astigmatism and anisometropia ≤ 1.50	Group 1 showed no significant differences in myopia progression (−0.52 ± 0.66 vs. −0.40 ± 0.72 D) and axial elongation (0.32 ± 0.26 vs. 0.28 ± 0.28 mm, both *p* > 0.05) between the first and the later 3 years. In the last 2.5 years, DIMS groups (Groups 1 and 3) had less myopia progression and axial elongation than the SVL groups (Groups 2 and 4). There was no evidence of rebound after stopping the treatment.
***Li et al. (2023)*** [25]	12 months	Retrospective cohort study	Data from 106 children wearing DIMS lenses with a 1-year follow-up were divided into two groups according to the increase in AL in one year: rapid (>0.2 mm) and slow (≤0.2 mm) axial elongation groups.	Cycloplegic autorefraction and AL were measured at baseline and after 6 and 12 months. The area of choriocapillaris flow voids and choroidal thickness at baseline were measured.	Chinese (Asian)	7–14 years	Myopia between −0.75 and −5.00 D; astigmatism and anisometropia ≤ 1.50	A smaller choriocapillaris flow voids area may slow myopia progression. For children wearing DIMS lenses, older age, initially less myopic eyes, larger pupil size, and steeper corneal curvature were protective factors for myopia control effects.
***Chun et al. (2024)*** [29]	31.98 ± 9.97 months	Retrospective, observational cohort study	Data from 489 and 156 patients who were prescribed DIMS and SVL, respectively, were collected. Patients with previous myopia control strategies were also included.	The changes in SER and AL were measured and normalized to annual changes. The correlation between age at baseline and annual change in AL was also examined.	Chinese (Asian)	3–17 years	Wearing duration of DIMS or SVL had to be at least 11.5 months	DIMS lenses could potentially reduce axial elongation, with the effect sustained with increased duration of lens wear. A small proportion of patients (2.7%) experienced a clinically significant axial shortening after wearing DIMS lenses more than 2 years.
***Neller et al. (2024)*** [30]	12 months	Retrospective, descriptive, non-interventional study	Data from 83 children were collected.	To monitor the efficacy of the myopia control strategies, a comparison between the patient’s annual AL growth rate with the average physiological AL growth rate of an age-matched cohort of emmetropic children was performed.	Caucasian (European)	6.4 to 15.2 years males; 7.2 to 16.9 females	Children with a 12-month follow-up; a minimum 10 months of continuous wear of DIMS spectacle lenses prior to 12-month follow-up	Treatment success regarding AL growth and myopia progression was achieved in 46% and 65%, respectively. Male eyes with moderate AL showed treatment success in a higher proportion compared to eyes with high AL; younger children showed treatment success in a lower proportion than older children.
***Buzzonetti et al. (2024)*** [31]	36 months	non-randomized experimenter-masked retrospective controlled observational study	Data from 80 participants were collected. Children were divided into four groups: patients wearing DIMS spectacle lenses older or younger than 10 years (group A and group C) and age-matched control groups (group B and group D) wearing SVL.	Cycloplegic SER and AL were measured at baseline and at 12-, 24-, and 36-month follow-ups.	Caucasian (European)	6–16 years	Myopia between −0.50 and −4.00 D; astigmatism ≤ 2 and anisometropia ≤ 1.	At 36 months, SER and AL increase were significantly reduced in groups A and C, respectively, compared to groups B and D. DIMS spectacles seem to slow myopia progression in pediatric patients with a major effectiveness in children older than 10 years of age.
***Domsa et al. (2024)*** [32]	12 months	Retrospective, observational study	The study included a cohort of 62 participants who were prescribed DIMS lenses following documented myopia progression of −0.50 D or more per year during prior SVL use	Cycloplegic SER, and AL were recorded at baseline, 6 months and 12 months. Information on family history of myopia was collected and participants were periodically asked to complete a quality of life questionnaire.	Caucasian (European)	4–17 years	Myopia between −0.875 and −8.75 D; astigmatism ≤ 3.25; –0.5 spherical D/year or more progression in the year before DIMS therapy.	At 12 months, 50% of patients showed no progression. Baseline astigmatism and younger age adversely affected therapy outcomes in both SER and AL, while severe maternal myopia led to greater SER progression. Patients reported consistent satisfaction with treatment, with minimal side effects, which diminished over the year.
***Fatimah et al. (2024)*** [35]	5 months	Cross-sectional qualitative study	A total of 29 interviews were performed, 15 with children and 14 with parents.	Separate in-depth interviews were conducted with children (mean age: 12.47 ± 2.13 years) and their parents based on prepared guides.	Indian (Asian)	12.47 ± 2.13 years (children)	Children who had used SVL in the past before using DIMS and had used it for a minimum of 1 month.	While participants were generally satisfied with DIMS lenses across social, physical, and psychological domains, concerns were noted regarding cost, accessibility, and environmental quality. Although parents observed few behavioral changes, children frequently reported adaptation-related symptoms such as blurred peripheral vision, eyestrain, and headaches.
***Wojtczak-Kwaśniewska et al. (2025)*** [33]	1 month	Randomized prospective study	A total of 21 participants were enrolled. Thirteen participants had low myopia (>−3.00 D), seven had medium myopia.	The study consisted of two parts: (I) examination of visual parameters and (II) visual evoked potential testing. (−3.00 to −6.00 D) and one had high myopia (<−6.00 D). The mean SER for all participants was −2.44 ± 1.60 D.	Caucasian (European)	20–30 years	Astigmatism ≤ 1.50 D, best-corrected visual acuity 0.00 logMAR or better, SER ≤ −0.50 D in at least one eye, normal binocular vision	No clinically significant differences in visual parameters or visual cortex responses between SVL and DIMS lenses after 2 weeks of adaptation. DIMS lenses produced slightly better high-contrast VA than SVL and a larger accommodative response. No significant differences in low-contrast VA, heterophoria, near point of convergence, stereopsis or contrast sensitivity were observed. The latencies and amplitudes of the early and late components of the visual evoked potentials did not differ significantly between lenses.
***Yahaya et al. (2025)*** [34]	24 months	Prospective, self-controlled study.	A total of 23 Malay myopic children were prescribed DIMS lenses and analyzed at baseline, 12, and 24 months.	Assessments included stereopsis, near point of convergence, phoria, positive/negative fusional vergence, amplitude of accommodation, accommodative lag, positive/negative relative accommodation, and accommodative convergence to accommodation ratio.	Malaysians (Asian)	7–15 years	SER: −0.50 to −5.00 D; astigmatism and anisometropia of ≤1.50 D; monocular best-corrected visual acuity of 6/6 or better; no previous myopia control treatment	Wearing DIMS lenses for 24 months resulted in changes in binocular vision and accommodation while slowing myopia progression.
***Li et al. (2025)*** [36]	12 months	Randomized double-blind prospective controlled clinical trial	176 myopic participants were randomly assigned into the DIMS group or the control SVL group.	Refractive error and AL measurements at baseline, three-, six-, nine-, and 12-month follow-up visits were monitored. The Quality of Life Impact of Refractive Correction questionnaire was used to evaluate the vision-related quality of life at baseline and at 12 months.	Chinese (Asian)	7–14 years	SER of −8.00∼0.00 D; astigmatism ≤ 1.50 D and anisometropia of ≤2.00 D; best-corrected visual acuity ≤ 0.0 LogMAR	The use of DIMS lenses in children was found to slow down myopia progression compared to SVL, without negatively affecting their overall quality of life. The mean differences in axial elongation and myopia progression were 0.13 mm and −0.28 D between the two groups. No significant difference in the Quality of Life Impact of Refractive Correction score was found between the two groups.

DIMS: Defocus Incorporated Multiple Segments; SVL: single-vision lenses; VA: visual acuity; SER: spherical equivalent refraction; AL: axial length; D: diopters.

**Table 2:** Clinical studies on therapeutic efficacy of Highly Aspherical Lenslet Target lenses.

Author (Year)	Duration	Type of Study	Population & Type of Lens	Assessment	Race	Age	Inclusion Criteria	Main Outcomes
***Bao et al. (2022*)** [37]	24 months	double-masked randomized clinical trial	157 participants were randomly assigned to receive spectacle lenses with HALT, SAL lenses, or SVL.	Changes in SER and AL and their differences between groups were evaluated.	Chinese (Asian)	8–13 years	SER of −0.75 D to −4.75 D and astigmatism with less than −1.50 D	HALT and SAL reduced the rate of myopia progression and axial elongation throughout 2 years, with higher efficacy for HALT. Longer wearing hours resulted in better myopia control efficacy for HALT.
***Bao et al. (2022)*** [38]	12 months	Randomized, controlled, double-masked study	170 children were randomized to receive HALT, SAL, or SVL.	Cycloplegic autorefraction, AL and best-corrected visual acuity were measured at baseline and 6-month intervals.	Chinese (Asian)	8–13 years	Myopia of −0.75 D to −4.75 D; astigmatism ≤ 1.50 D and anisometropia of ≤1.00 D.	HALT lenses effectively slowed myopia progression and axial elongation compared to SVL.
***Sankaridurg et al. (2022)*** [39]	12 months	Prospective, double-blind, single-center, randomized, cross-over trial.	119 children were randomized to wear either HALT or SVL, and after 6 months crossed over to the other lens for another 6 months. At the end, both groups wore HALT for a further 6 months.	The main outcome measures were a comparison between HALT and SVL for change in SER and AL during each stage.	Vietnamese (Asian)	8–13 years	SER of −0.75 to −4.75 D; astig- Matism ≤ −1.50 D, anisometropia of ≤1.00 D, Visualacuity of ≥0.05 logMAR	Comparisons indicated that HALT lenses can slow myopia. Children were compliant with lens wearing, and data were not suggestive of rebound effect when patients were switched from HALT to SVL.
***Gao et al. (2022)*** [43]	/	Randomized Trial	Participants were recruited through an internal subject database and word-of-mouth. Twenty-one participants volunteered in this study.	Automated static perimetry was employed to measure the visual field sensitivity. Targets were white light dots of various luminance levels and size 0.43°, randomly appearing at 76 locations within 30° eccentricity.	Singaporeans (Asian)	21–65 years	Refractive error of sphere between −10 and +10 D, and astigmatism not more than 1 D.	HALT lenses did not change detection sensitivity to static targets in the whole visual field within 30° eccentricity.
***Drobe et al. (2023*)** [40]	48 months	Clinical trial extension	44 children who wore HALT for 3 years, accepted to be followed for two more years.	SER of cycloplegic autorefraction and AL were measured at the end of year 4. Change of AL with HALT was compared to a SVL model based on SVL data of the first two years of the same clinical trial	Asian	11–15 years	Asian children who wore HALT lenses for 3 years and agreed to follow-up for another 2 years	Myopia progression and axial elongation in children wearing HALT lenses were slower than in a modeled control SVL group during year 4.
***Huang et al. (2023)*** [42]	24 months	Prospective, randomized, controlled, and double-blind trial	170 children were included. Participants were randomized to wear HALT, SAL, or SVL	Peripheral eye length and peripheral refraction changes were measured at 0° central and 15° and 30° in the nasal and temporal retina every 6 months for 2 years.	Chinese (Asian)	8–13 years	SER from 0.75 to −4.75 D, astigmatism ≤1.50 D, anisometropia ≤1.00 D, no strabismus or ocular diseases, no prior myopia control.	Participants with HALT exhibited faster peripheral eye elongation, leading to a flattened retina and a reduction in peripheral hyperopic defocus. In contrast, SVL and SAL groups showed retinal steepening and increased peripheral hyperopic defocus with myopia progression.
***Wong et al. (2024)*** [46]	24 months	Double-masked randomized clinical trial	170 children were randomly assigned to the HALT, SAL, or SVL groups	Axial elongation compared to eye growth patterns in non-myopes was measured	Chinese (Asian)	8–13 years	SE between −0.75 and −4.75 D, astigmatism ≤ 1.50 D, anisometropia ≤ 1.00 D and best-corrected visual acuity of 0.05 logMAR or better in each eye.	90% of children in the HALT group achieved axial growth rates that were similar to or slower than those expected in non-myopic children.
***Wang et al. (2025)*** [45]	12 months	Retrospective study	105 non-myopic children wore plano HALT spectacle lenses.	Efficacy was evaluated with pre-treatment rates acting as controls, and differences in changes over time were calculated.	Chinese (Asian)	4–9 years	astigmatism ≤ 0.75 D, best-corrected visual acuity equivalent or better than 6/7.5. No previous myopia control strategies were used.	Plano HALT lenses were effective in slowing axial elongation and SER progression among non-myopic children.
***Zhang et al. (2025)*** [44]	12 months	Randomized controlled trial	108 children were randomly assigned in a 1:1 ratio to wear either HALT or SVL.	Cycloplegic refraction, AL, and uncorrected visual acuity were measured at baseline, 6 months, and 12 months. Lens wearing time was objectively monitored using a wearable sensor device attached to the spectacle frames and subjectively recorded through guardian questionnaires at each follow-up.	Chinese (Asian)	6–9.9 years	SER from 0.00 to +2.00 D, refractive astigmatism ≤ 1.25 D, anisometropia ≤ 1.00 D, uncorrected visual acuity of 0.10 LogMAR or better in each eye, and willingness to consistently wear the prescribed spectacle lenses throughout the study period.	After 1 year, SER changes were similar between HALT and SVL groups. HALT lenses reduced AL elongation, especially in children wearing them over 30 h per week. AL and SER changes in the HALT group correlated with wearing time, suggesting HALT lenses are effective for low hyperopic children with high compliance.
***Li X et al. (2025)*** [41]	60 months	Randomized Controlled Trial for the first 2 years. Prospective cohort extension study for the subsequent 3 years (Years 3–5)	Children were randomized to HALT, SAL, or SVL lenses Fifty-two HALT wearers entered a 1-year extension, and 44 completed 5 years of HALT use. An extrapolated SVL control group was created from literature data.	SER and AL were measured each year.	Chinese (Asian)	8–13 years	SER of −0.75 D to −4.75 D and astigmatism with less than −1.50 D	HALT lenses reduced myopia progression and axial elongation over 5 years compared to the SVL group, also lowering the incidence of high myopia

HALT: Highly Aspherical Lenslet Target; SAL: slightly aspherical lenslets; SVL: single-vision lenses; SER: spherical equivalent refraction; AL: axial length; D: diopters.

**Table 3:** Clinical studies on the efficacy of Cylindrical Annular Refractive Elements lenses for myopia progression.

Author (Year)	Duration	Type of Study	Population & Type of Lens	Assessment	Race	Age	Inclusion Criteria	Main Outcomes
***Liu et al. (2023)*** [48]	12 months	Randomized controlled study	96 children were included in the analysis (52 in CARE lenses group and 44 in the SV lenses group)	Cycloplegic autorefraction SER and AL were measured at baseline and 6-month intervals. Adaptation and compliance questionnaires were administered during all visits.	Chinese (Asian)	8–12 years	SER of −1.00 D to −4.00 D; astigmatism < 1.50 D cylinder; absence of ocular pathology and systemic disease; no history of ocular surgery; no use of myopia control measures in the past 6 months.	Adjusted 1-year myopia progression was −0.56 D for CARE and −0.71 D for single-vision spectacle lenses. Adjusted 1-year eye growth was 0.27 mm for CARE and 0.35 mm for single vision.
***Chen et al. (2024)*** [49]	12 months	Prospective, double-masked, multi-centre clinical trial	240 children randomized to one of three groups of 80 participants: single-vision spectacle lens, CARE lenses (7 mm central clear zone surrounded by treatment zone incorporating CARE with mean surface power of +4.6 D) and CARE S (9 mm central clear zone surrounded by treatment zone comprising CARE with mean surface power of +3.8 D)	Cycloplegic SE and AL were measured at 6-month intervals.	Chinese (Asian)	6–13 years	Refractive error ranging from −0.75 D to −5.00 with astigmatism ≤ 1.50 D; anisometropia of ≤1.50 D; best corrected visual acuity of ≥1.0 in both eyes; absence of ocular pathology and systemic disease; no history of ocular surgery; no use of myopia control measures in the past 3 months.	Changes in SER and axial length were significantly different between the groups at both 6 and 12 months. Progression was slower with CARE and CARE S compared to single-vision lenses but did not differ from each other.
***Chen et al. (2025)*** [50]	24 months	Prospective, double-masked, multicenter, randomized clinical trial	240 children randomized to one of three groups of 80 participants: single-vision spectacle lens, CARE lenses (7 mm central clear zone surrounded by treatment zone incorporating CARE with mean surface power of +4.6 D) and CARE S (9 mm central clear zone surrounded by treatment zone comprising CARE with mean surface power of +3.8 D)	Cycloplegic SE and AL were measured at 6-month intervals	Chinese (Asian)	6–13 years	Refractive error ranging from −0.75 D to −5.00 with astigmatism ≤ 1.50 D; anisometropia of ≤1.50 D; best corrected visual acuity of ≥1.0 in both eyes; absence of ocular pathology and systemic disease; no history of ocular surgery; no use of myopia control measures in the past 3 months.	Myopia progression was significantly slower with both CARE lenses (−0.73 ± 0.63 D/0.40 ± 0.26 mm) and CARE S lenses (−0.80 ± 0.56 D/0.44 ± 0.25 mm) compared to single-vision lenses. Progression did not differ significantly between CARE lenses.
***Alvarez-Peregrina et al. (2025)*** [51]	12 months	Randomized, parallel-group, double-masked, multicenter clinical trial	226 children (117 and 109 wearing the single-vision lenses and CARE lenses, respectively)	AL and SER were measured at baseline, 6 and 12 months. Wearability questionnaires were administered at 1 week and 3 months. Central and peripheral visual acuity was recorded at dispensing and after 3 months.	Caucasian (European)	6–13 years	Best-corrected monocular and binocular visual acuity of 0.00 logMAR or better, cycloplegic spherical equivalent between −0.75 D and −5.00 D in both eyes, astigmatism of −1.50 D or less, anisometropia of 1.00 D or less. A myopia progression of at least 0.50 D in the year preceding enrolment in the trial. Absence of ocular and systemic diseases; no history of ocular surgery; no use of myopia control strategies.	Children wearing CARE lenses showed less myopia progression, with a difference in SER and axial length progression (compared to single-vision lenses) of −0.21 D and 0.14 mm, respectively. Central visual acuity did not decrease with CARE lenses. Analysis of fast progressors indicated that 39.7% of single-vision lenses progressed by ≤−0.50 D/year compared to 21.1% with CARE. For axial length, 56.0% of single-vision lenses users had an elongation ≥0.20 mm compared to 21.3% with CARE.

CARE: Cylindrical Annular Refractive Elements; SER: spherical equivalent refraction; D: diopters.

**Table 4:** Clinical studies on comparison of therapeutic efficacy among Defocus Incorporated Multiple Segments lenses, Highly Aspherical Lenslet Target lenses and Cylindrical Annular Refractive Elements lenses.

Author (Year)	Duration	Type of Study	Population & Type of Lens	Assessment	Race	Age	Inclusion Criteria	Main Outcomes
***Guo et al. (2023)***[52]	12 months	Retrospective cohort study	A total of 257 children were included in the analysis (193 in the HALT group and 64 in the DIMS group).	Standardized 1-year changes in SER and AL were calculated from baseline for all participants with at least one year of follow-up.	Chinese (Asian)	Younger than 16 years	Children, without strabismus, amblyopia, or other ocular or systematic abnormalities.	Children wearing HALT lenses had less myopia progression and axial elongation than those wearing DIMS lenses.
***Lembo et al. (2024)***[54]	24 months	Retrospective cohort study	146 participants wore either DIMS (73) or HALT (73) spectacle lenses for a minimum of two years.	AL and SER were measured at baseline, 1 year, and 2 years.	Caucasian (European)	6–17 years	Children with progressive myopia (SER ≤ −0.50 D), who wore either DIMS or HALT spectacle lenses continuously for two years and completed both 1- and 2-year follow-up visits.	Differences were neither clinically nor statistically significant, except for a slightly higher AL increase with DIMS at 1 year. 38.4% of DIMS users showed no SER progression at 2 years compared to 21.9% for HALT users.
***Najji et al. (2025)***[53]	36 months	Longitudinal, retrospective, comparative, observational, real-world study	The study included three groups, each comprising 2542 children with comparable follow-up durations. The treated group was prescribed myopia control spectacles (DIMS, *n* = 1786); HALT, *n* = 585; both, *n* = 171) during the follow-up period, while the two comparison groups continued wearing SVL throughout.	The difference in myopia progression was calculated between SVL groups and the MCS group. DIMS and HAL were also compared for myopia progression.	Caucasian (European)	4–15 years	Baseline refractive error of −0.5 D or lower. The SVL group of children had to have at least three lenses prescriptions, with one prescription taken between 12 and 18 months after baseline of the study. For the DIMS + HALT group, the children had to have received at least two SVL prescriptions in the pre-switch phase and then having switched to myopia control spectacles for the remainder of the follow-up (post-switch phase).	Both DIMS and HALT lenses demonstrated efficacy in reducing myopia progression. While a statistically significant lower myopia progression rate was observed in the HALT group, this difference was not clinically significant. DIMS and HALT are also able to reduce myopia progression among younger children aged 4 to 6 years.
***Gupta et al. (2025)***[55]	12 months	Prospective, interventional, double-blinded, randomized clinical trial.	120 children were randomly assigned (1:1:1) to wear either DIMS, HALT or CARE lenses full-time.	Cycloplegic refraction and AL measurements were taken at baseline and after 1 year. The primary outcome was the change in the rate of myopia progression	Indian (Asian)	5–15 years	Myopia progression of ≥0.5 D/year; refractive error between −1 D and −8 D; best- corrected visual acuity of 6/9 or better in both eyes.	DIMS, HALT, and CARE lenses were all effective in significantly reducing the rate of myopia progression, with no adverse effects reported. Among the three designs, DIMS and HALT demonstrated comparable efficacy, both outperforming CARE lenses at the 1-year follow-up.

HALT: Highly Aspherical Lenslet Target; DIMS: Defocus Incorporated Multiple Segments; SER: spherical equivalent refraction; AL: axial length; D: diopters; CARE: Cylindrical Annular Refractive Elements; SVL: single-vision lenses.

## Data Availability

Not applicable.

## References

[1] Holden B.A., Fricke T.R., Wilson D.A., Jong M., Naidoo K.S., Sankaridurg P., Wong T.Y., Naduvilath T.J., Resnikoff S. (2016). Global Prevalence of Myopia and High Myopia and Temporal Trends from 2000 through 2050. Ophthalmology.

[2] Ruiz-Medrano J., Montero J.A., Flores-Moreno I., Arias L., Garcia-Layana A., Ruiz-Moreno J.M. (2019). Myopic maculopathy: Current status and proposal for a new classification and grading system (ATN). Prog. Retin. Eye Res..

[3] Wildsoet C.F., Chia A., Cho P., Guggenheim J.A., Polling J.R., Read S., Sankaridurg P., Saw S.-M., Trier K., Walline J.J. (2019). IMI–interventions for controlling myopia onset and progression report. Investig. Ophthalmol. Vis. Sci..

[4] Gifford K.L., Richdale K., Kang P., Aller T.A., Lam C.S., Liu Y.M., Michaud L., Mulder J., Orr J.B., Rose K.A. (2019). IMI–clinical management guidelines report. Investig. Ophthalmol. Vis. Sci..

[5] Morgan I.G., French A.N., Ashby R.S., Guo X., Ding X., He M., Rose K.A. (2018). The epidemics of myopia: Aetiology and prevention. Prog. Retin. Eye Res..

[6] Neitz J., Kuchenbecker J., Neitz M. (2020). Ophthalmic Lenses Treat Myopia. https://patents.google.com/patent/WO2018026697A1/en.

[7] Wu P.C., Chen C.T., Lin K.K., Sun C.C., Kuo C.N., Huang H.M., Poon Y.C., Yang M.L., Chen C.Y., Huang J.C. (2018). Myopia Prevention and Outdoor Light Intensity in a School-Based Cluster Randomized Trial. Ophthalmology.

[8] Williams K.M., Bertelsen G., Cumberland P., Wolfram C., Verhoeven V.J., Anastasopoulos E., Buitendijk G.H., Cougnard-Gregoire A., Creuzot-Garcher C., Erke M.G. (2015). Increasing Prevalence of Myopia in Europe and the Impact of Education. Ophthalmology.

[9] VanderVeen D.K., Kraker R.T., Pineles S.L., Hutchinson A.K., Wilson L.B., Galvin J.A., Lambert S.R. (2019). Use of orthokeratology for the prevention of myopic progression in children: A report by the American Academy of Ophthalmology. Ophthalmology.

[10] Huang J., Wen D., Wang Q., McAlinden C., Flitcroft I., Chen H., Saw S.M., Chen H., Bao F., Zhao Y. (2016). Efficacy Comparison of 16 Interventions for Myopia Control in Children: A Network Meta-analysis. Ophthalmology.

[11] Maulvi F.A., Desai D.T., Kalaiselvan P., Shah D.O., Willcox M.D.P. (2025). Current and emerging strategies for myopia control: A narrative review of optical, pharmacological, behavioural, and adjunctive therapies. Eye.

[12] Yap T.P., Mishu M.P. (2024). Pharmaceutical Prescribing Privileges for Optometrists to Combat Childhood Myopia in Singapore: Public Health Policy Review and Analysis. Children.

[13] Perea-Romero J., Signes-Soler I., Badenes-Ribera L., Tauste A. (2025). Efficacy of spectacle lenses specifically designed for myopia control: Systematic review and meta-analysis. Graefes. Arch. Clin. Exp. Ophthalmol..

[14] Erdinest N., London N., Lavy I., Berkow D., Landau D., Morad Y., Levinger N. (2023). Peripheral Defocus and Myopia Management: A Mini-Review. Korean J. Ophthalmol..

[15] Arumugam B., Hung L.-F., To C.-H., Holden B., Smith E.L. (2014). The Effects of Simultaneous Dual Focus Lenses on Refractive Development in Infant Monkeys. Investig. Ophthalmol. Vis. Sci..

[16] McFadden S.A., Tse D.Y., Bowrey H.E., Leotta A.J., Lam C.S., Wildsoet C.F., To C.-H. (2014). Integration of Defocus by Dual Power Fresnel Lenses Inhibits Myopia in the Mammalian Eye. Investig. Ophthalmol. Vis. Sci..

[17] Arumugam B., Hung L.-F., To C.-H., Sankaridurg P., Smith E.L. (2016). The Effects of the Relative Strength of Simultaneous Competing Defocus Signals on Emmetropization in Infant Rhesus Monkeys. Investig. Ophthalmol. Vis. Sci..

[18] Carlà M.M., Boselli F., Giannuzzi F., Gambini G., Caporossi T., De Vico U., Savastano A., Baldascino A., Rizzo C., Kilian R. (2022). Overview on Defocus Incorporated Multiple Segments Lenses: A Novel Perspective in Myopia Progression Management. Vision.

[19] Lam C.S.Y., Tang W.C., Tse D.Y.-Y., Lee R.P.K., Chun R.K.M., Hasegawa K., Qi H., Hatanaka T., To C.H. (2020). Defocus Incorporated Multiple Segments (DIMS) Spectacle Lenses Slow Myopia Progression: A 2-Year Randomised Clinical Trial. Br. J. Ophthalmol..

[20] Lu Y., Lin Z., Wen L., Gao W., Pan L., Li X., Yang Z., Lan W. (2020). The Adaptation and Acceptance of Defocus Incorporated Multiple Segment Lens for Chinese Children. Am. J. Ophthalmol..

[21] Zhang H.Y., Lam C.S.Y., Tang W.C., Leung M., To C.H. (2020). Defocus Incorporated Multiple Segments Spectacle Lenses Changed the Relative Peripheral Refraction: A 2-Year Randomized Clinical Trial. Investig. Ophthalmol. Vis. Sci..

[22] Zhang H.Y., Lam C.S.Y., Tang W.C., Lee P.H., Tse D.Y., To C.H. (2023). Changes in Relative Peripheral Refraction in Children Who Switched from Single-Vision Lenses to Defocus Incorporated Multiple Segments Lenses. Ophthalmic Physiol. Opt..

[23] Zhang H., Lam C.S.Y., Tang W.-C., Leung M., Qi H., Lee P.H., To C.-H. (2022). Myopia Control Effect Is Influenced by Baseline Relative Peripheral Refraction in Children Wearing Defocus Incorporated Multiple Segments (DIMS) Spectacle Lenses. J. Clin. Med..

[24] Chun R.K.M., Zhang H., Liu Z., Tse D.Y.Y., Zhou Y., Lam C.S.Y., To C.H. (2023). Defocus Incorporated Multiple Segments (DIMS) Spectacle Lenses Increase the Choroidal Thickness: A Two-Year Randomized Clinical Trial. Eye Vis..

[25] Li X., Hu J., Peng Z., Chen S., Sun L., Wang K., Li Y., Zhao M. (2023). Association between Choriocapillaris Perfusion and Axial Elongation in Children Using Defocus Incorporated Multiple Segments (DIMS) Spectacle Lenses. Eye.

[26] Lam C.S.Y., Tang W.C., Qi H., Radhakrishnan H., Hasegawa K., To C.H., Charman W.N. (2020). Effect of Defocus Incorporated Multiple Segments Spectacle Lens Wear on Visual Function in Myopic Chinese Children. Transl. Vis. Sci. Technol..

[27] Lam C.S., Tang W.C., Lee P.H., Zhang H.Y., Qi H., Hasegawa K., To C.H. (2022). Myopia Control Effect of Defocus Incorporated Multiple Segments (DIMS) Spectacle Lens in Chinese Children: Results of a 3-Year Follow-up Study. Br. J. Ophthalmol..

[28] Lam C.S.Y., Tang W.C., Zhang H.Y., Lee P.H., Tse D.Y.Y., Qi H., Vlasak N., To C.H. (2023). Long-Term Myopia Control Effect and Safety in Children Wearing DIMS Spectacle Lenses for 6 Years. Sci. Rep..

[29] Chun R.K.M., Wong K.Y.Q., Lam C.S.Y., To C.-H., Liu K.K.K., Wong Y.-Z., Tang W.-C., Chan N., Kwok D., Cheung M. (2024). Real-World Outcomes of Defocus Incorporated Multiple Segments Lenses on Retarding Axial Elongation in Myopic Children and Adolescents. Front. Med..

[30] Neller B., Neller K., Schwahn H., Mattern A.-I., Devenijn M., Langenbucher A., Seitz B., Kaymak H. (2024). Effect of Defocus Incorporated Multiple Segments (DIMS) Spectacle Lenses on Myopia Progression in Children: A Retrospective Analysis in a German Real-Life Clinical Setting. BMC Ophthalmol..

[31] Buzzonetti L., Petroni S., Federici M., Valente P., Iarossi G. (2024). Effectiveness of Defocus Incorporated Multiple Segments in Slowing Myopia Progression in Pediatric Patients as a Function of Age: Three-Year Follow-Up. Diseases.

[32] Domsa P., Bankó É.M., Körtvélyes J., Meigen C., Széchey R., Lantos K., Nagy Z.Z., Csutak A. (2024). Astigmatism and Maternal Myopia as Important Factors Affecting Success Rate of DIMS Lens Treatment. BMJ Open Ophthalmol..

[33] Wojtczak-Kwaśniewska M., Domagalski M., Dymczyk M., Padurska M., Przekoracka K., Przekoracka-Krawczyk A. (2025). Do Myopia Control Spectacle Lenses with Defocus Incorporated Multiple Segments Technology Alter Visual Parameters and Cortical Activity?. Ophthalmic Physiol. Opt..

[34] Yahaya N.A., Mohd-Ali B., Norazman F.N.N., Syed Mohd Dardin S.F., Mohamad Shahimin M., Mohamad Fadzil N. (2025). Assessment of Binocular Vision and Accommodation in Myopic Children Wearing Defocus Incorporated Multiple Segments (DIMS) Spectacle Lenses for 24 Months. J. Optom..

[35] Fatimah M., Agarkar S., Narayanan A. (2024). Impact of Defocus Incorporated Multiple Segments (DIMS) Spectacle Lenses for Myopia Control on Quality of Life of the Children: A Qualitative Study. BMJ Open Ophthalmol..

[36] Li X., Ma W., Song Y., Yap M., Liu L. (2025). Comparison of Myopic Progression and Quality of Life Wearing Either DIMs Lenses or Single-Vision Myopia Correcting Spectacles. J. Ophthalmol..

[37] Bao J., Huang Y., Li X., Yang A., Zhou F., Wu J., Wang C., Li Y., Lim E.W., Spiegel D.P. (2022). Spectacle Lenses with Aspherical Lenslets for Myopia Control vs Single-Vision Spectacle Lenses: A Randomized Clinical Trial. JAMA Ophthalmol..

[38] Bao J., Yang A., Huang Y., Li X., Pan Y., Ding C., Lim E.W., Zheng J., Spiegel D.P., Drobe B. (2022). One-Year Myopia Control Efficacy of Spectacle Lenses with Aspherical Lenslets. Br. J. Ophthalmol..

[39] Sankaridurg P., Weng R., Tran H., Spiegel D.P., Drobe B., Ha T., Tran Y.H., Naduvilath T. (2023). Spectacle Lenses with Highly Aspherical Lenslets for Slowing Myopia: A Randomized, Double-Blind, Cross-Over Clinical Trial: Parts of These Data Were Presented as a Poster at the Annual Research in Vision and Ophthalmology Meeting, 2022. Am. J. Ophthalmol..

[40] Drobe B., Xue L., Huang Y., Lim E.W., Bao J. (2023). Spectacle Lenses with Highly Aspherical Lenslets for Myopia Control: 4-Year Clinical Trial Results. Investig. Ophthalmol. Vis. Sci..

[41] Li X., Huang Y., Liu C., Chang X., Cui Z., Yang Q., Drobe B., Bullimore M.A., Chen H., Bao J. (2025). Myopia Control Efficacy of Spectacle Lenses with Highly Aspherical Lenslets: Results of a 5-Year Follow-up Study. Eye Vis..

[42] Huang Y., Zhang J., Yin Z., Yang A., Spiegel D.P., Drobe B., Chen H., Bao J., Li X. (2023). Effects of Spectacle Lenses with Aspherical Lenslets on Peripheral Eye Length and Peripheral Refraction in Myopic Children: A 2-Year Randomized Clinical Trial. Transl. Vis. Sci. Technol..

[43] Gao Y., Spiegel D.P., Muzahid I.A.I., Lim E.W., Drobe B. (2022). Spectacles with Highly Aspherical Lenslets for Myopia Control Do Not Change Visual Sensitivity in Automated Static Perimetry. Front. Neurosci..

[44] Zhang Z., Zeng L., Gu D., Wang B., Kang P., Watt K., Zhou J., Zhou X., Chen Z., Yang D. (2025). Spectacle Lenses with Highly Aspherical Lenslets for Slowing Axial Elongation and Refractive Change in Low-Hyperopic Chinese Children: A Randomized Controlled Trial. Am. J. Ophthalmol..

[45] Wang L., Wong Y.L., Drobe B., Wang X. (2025). Effectiveness of Spectacle Lenses with Highly Aspherical Lenslets in Slowing Axial Elongation among Non-Myopic Children. Clin. Exp. Optom..

[46] Wong Y.L., Li X., Huang Y., Yuan Y., Ye Y., Lim E.W., Yang A., Spiegel D., Drobe B., Bao J. (2024). Eye Growth Pattern of Myopic Children Wearing Spectacle Lenses with Aspherical Lenslets Compared with Non-Myopic Children. Ophthalmic Physiol. Opt..

[47] Alvarez-Peregrina C., Sanchez-Tena M.A., Martinez-Perez C., Villa-Collar C., Ohlendorf A., Clinical Evaluation of MyoCare in Europe—The CEME Study Group (2023). Clinical Evaluation of MyoCare in Europe (CEME): Study protocol for a prospective, multicenter, randomized, double-blinded, and controlled clinical trial. Trials.

[48] Liu X., Wang P., Xie Z., Sun M., Chen M., Wang J., Huang J., Chen S., Chen Z., Wang Y. (2023). One-year myopia control efficacy of cylindrical annular refractive element spectacle lenses. Acta Ophthalmol..

[49] Chen X., Wu M., Yu C., Ohlendorf A., Rifai K., Boeck-Maier C., Wahl S., Yang Y., Zhu Y., Li L. (2024). Slowing myopia progression with cylindrical annular refractive elements (CARE) spectacle lenses-Year 1 results from a 2-year prospective, multi-centre trial. Acta Ophthalmol..

[50] Chen X., Wu M., Yu C., Ohlendorf A., Li W., Liu N., Yang Y., Li L., Sankaridurg P. (2025). Efficacy of Cylindrical Annular Refractive Elements (CARE) Spectacle Lenses in Slowing Myopia Progression Over 2 Years. Am. J. Ophthalmol..

[51] Alvarez-Peregrina C., Sanchez-Tena M.A., Villa-Collar C., Martinez-Perez C., de Corcuera-Terrero B., Liu N., Li W., Sankaridurg P., Ohlendorf A., Clinical Evaluation of MyoCare in Europe—The CEME Study Group (2025). Clinical evaluation of MyoCare in Europe (CEME) for myopia management: One-year results. Ophthalmic Physiol. Opt..

[52] Guo H., Li X., Zhang X., Wang H., Li J. (2023). Comparing the Effects of Highly Aspherical Lenslets versus Defocus Incorporated Multiple Segment Spectacle Lenses on Myopia Control. Sci. Rep..

[53] Najji R., Bullimore M., Resnikoff S., Bron A.M., Naudin M., Giraud C., Bourne R., Jonas J.B., Leveziel N. (2025). The Real-World Effectiveness of Defocus Incorporated Multiple Segments and Highly Aspherical Lenslets on Myopia Control: A Longitudinal Study from the French Myopia Cohort. BMJ Open Ophthalmol..

[54] Lembo A., Schiavetti I., Serafino M., Caputo R., Nucci P. (2024). Comparison of the Performance of Myopia Control in European Children and Adolescents with Defocus Incorporated Multiple Segments (DIMS) and Highly Aspherical Lenslets (HAL) Spectacles. BMJ Paediatr. Open.

[55] Gupta V., Saxena R., Dhiman R., Phuljhele S., Sharma N. (2025). Comparative Evaluation of Different (Peripheral Defocus Based) Spectacle Designs in Preventing Myopia Progression: A Double-Blinded Randomised Clinical Trial. Ophthalmic Physiol. Opt..

